# miRNAs in the Expression Regulation of Dopamine-Related Genes and Proteins in Endometrial Cancer

**DOI:** 10.3390/jcm10214939

**Published:** 2021-10-26

**Authors:** Michał Czerwiński, Anna Bednarska-Czerwińska, Nikola Zmarzły, Dariusz Boroń, Marcin Oplawski, Beniamin Oskar Grabarek

**Affiliations:** 1 American Medical Clinic, 40-600 Katowice, Poland; 2 Gyncentrum Fertility Clinic, 40-121 Katowice, Poland; czerwinskaa002@gmail.com; 3 Faculty of Medicine, University of Technology in Katowice, 41-800 Zabrze, Poland; 4 Department of Histology, Cytophysiology and Embryology, Faculty of Medicine, University of Technology in Katowice, 41-800 Zabrze, Poland; nikola.zmarzly@gmail.com (N.Z.); dariusz@boron.pl (D.B.); bgrabarek7@gmail.com (B.O.G.); 5 Department of Gynecology and Obstetrics with Gynecologic Oncology, Ludwik Rydygier Memorial Specialized Hospital, 31-826 Kraków, Poland; marcin.oplawski@gmail.com; 6 Department of Gynecology and Obstetrics, TOMMED Specjalisci od Zdrowia, Fredry 22, 40-662 Katowice, Poland

**Keywords:** endometrial cancer, dopamine, dopamine receptors, miRNAs

## Abstract

Disruption of the dopaminergic system leads to many diseases, including cancer. Dopamine and its receptors are involved in the regulation of proliferation, cell death, invasion, and migration. Better understanding of the mechanisms involved in these processes could reveal new molecular markers and therapeutic targets. The aim of this study was to determine the expression profile of dopamine-related genes and proteins in endometrial cancer and to assess whether miRNAs are involved in its regulation. Sixty women were recruited for the study: 30 with endometrial cancer and 30 without cancer. The expression profiles of dopamine-related genes were determined in endometrial tissue samples using microarrays and qRT-PCR. Then, protein concentration was determined with the ELISA test. In the last step, miRNA detection was performed using microarrays. The matching of miRNAs to the studied genes was carried out using the TargetScan tool. The analysis showed DRD2 and DRD3 overexpression, with a reduction in DRD5 expression, which could be due to miR-15a-5p, miR-141-3p, miR-4640-5p, and miR-221-5p activity. High levels of OPRK1 and CXCL12, related to the activity of miR-124-3p.1 and miR-135b-5p, have also been reported. Low COMT expression was probably not associated with miRNA regulation in endometrial cancer.

## 1. Introduction

Dopamine belongs to the catecholamine family and is a precursor of norepinephrine and epinephrine. It acts as a neurotransmitter in the central nervous system (CNS) and participates in the regulation of mood, behavior, cognition, addiction, and the reward system [1]. Dopamine is also produced peripherally, where it functions as a circulating hormone, modulating the immune response, blood pressure, and kidney function [2]. Dopamine activity depends on binding to its five receptors (D1–D5). Dopamine receptors (DR) can be divided into two groups according to their effect on adenylyl cyclase: activating (DRD1 and DRD5) and inhibiting (DRD2, DRD3, and DRD4) [3]. Moreover, the receptors also differ in their binding affinity within these groups. DRD5 has a 10-fold higher affinity than DRD1, while DRD3 and DRD4 bind dopamine similarly, but more strongly than DRD2 [4].

Disruption of the dopaminergic system leads to many diseases, including schizophrenia [5], Alzheimer’s disease [6], Parkinson’s disease [7], and cancer [8]. Studies on cancer biology show that dopamine receptors may be potential therapeutic targets due to their involvement in the regulation of proliferation, cell death, invasion, and migration [9]. However, the expression pattern of DR varies with the tumor type, indicating the need for further understanding of the importance of the dopaminergic system in tumor physiology [10].

Endometrial cancer is one of the most common gynecological cancers in the world [11]. It is diagnosed mainly in peri- and post-menopausal women; however, about 20–25% of cases arise earlier [12]. Endometrial cancer can be classified into two main types [13], as well as due to cancer staging [14] and grading [15], but such divisions are insufficient. For this reason, classification systems that also take into account molecular characteristics of the tumor are being established. The Cancer Genome Atlas (TCGA) Research Network’s goal was to eliminate over- or under-treatment by increasing diagnostic precision. Depending on the genetic characteristics, four subgroups have been distinguished: polymerase epsilon (POLE)-ultramutated, microsatellite instability-hypermutated, copy-number low, and copy-number high [16]. While promising, this classification is associated with high costs and technical difficulties, which makes it hard to incorporate into routine diagnostics. The Proactive Molecular Risk Classifier for Endometrial Cancer (ProMisE) has emerged as an alternative option to overcome these limitations. Immunohistochemical markers have been proposed as a substitute for sequencing. However, an alternative marker has not yet been found for all TCGA molecular groups, indicating the need for further research on this topic. [17].

Studies identifying novel molecular markers and potential therapeutic targets in cancer are also very promising [18]. They include microRNAs (miRNAs), belonging to the group of non-coding RNAs that regulate the expression of many genes. As a result, miRNAs participate in the modulation of important cellular processes, including metabolism, proliferation, apoptosis, migration, and differentiation. Depending on the context, activated signaling pathways may favor tumor progression, survival, metastasis, and epithelial-mesenchymal transition [19].

The aim of this study was to determine the expression profile of dopamine-related genes and proteins in endometrial cancer and to assess whether miRNAs are involved in its regulation.

## 2. Materials and Methods

The Bioethical Committee operating at the Regional Medical Chamber in Kraków approved the following study (185/KBL/OIL/202 and 186/KBL/OIL/2020). All procedures were performed in accordance with the guidelines of the 2013 Declaration of Helsinki. Written informed consent was obtained from all study participants.

### 2.1. Patients

A total of 60 women were recruited for the study, including 30 with endometrioid endometrial cancer confirmed by histopathological examination (study group) and 30 without neoplastic changes (control group). All patients were qualified for hysterectomy and treated at the Department of Gynecology and Obstetrics with Gynecologic Oncology at the Ludwik Rydygier Memorial Specialized Hospital. Endometrial tissue and blood samples were collected from the patients. According to the degree of histological differentiation, the following subgroups can be distinguished in the collected EC samples: G1, 15 cases; G2, 8 cases; and G3, 7 cases.

Exclusion criteria from the study group involved the diagnosis of non-endometrioid endometrial cancer, coexisting cervical cancer, and history of other types of cancer. Patients with endometriosis or adenomyosis, extreme obesity (Body Mass Index; BMI > 40), and using hormone therapy within the 24 months prior surgery were also excluded. In addition, patients included in the study were over 45 years old and after childbearing period.

Whole blood was collected using PAXgene Blood RNA Tubes. Endometrial tissue samples were placed in Eppendorf tubes with Allprotect Tissue reagent (Qiagen, Hilden, Germany, Cat No./ID: 76405). All samples were stored according to the manufacturer’s recommendations.

### 2.2. RNA Extraction

Total RNA extraction from whole blood was carried out using the PAXgene Blood RNA Kit (Invitrogen Life Technologies, Carlsbad, CA, USA, Cat No: 762174). RNA extraction from endometrial tissue samples was performed with TRIzol reagent (Invitrogen Life Technologies, Carlsbad, CA, USA, Cat No. 15596026). Agarose electrophoresis and spectrophotometry were used for qualitative and quantitative evaluation of the obtained extracts.

### 2.3. Microarray Analysis

The expression profile of dopamine-related genes was assessed using HG-U133A 2.0 microarrays (Affymetrix, Santa Clara, CA, USA), the GeneChip™ 3′IVT PLUS, and Ge-neChip™ HT 3′IVT PLUS Reagent kits (ThermoFisher Scientific, Waltham, MA USA, Cat No. 902416, 902417). Fluorescence intensity was measured with the Gene Array scanner (Agilent Technologies, Santa Clara, CA, USA). The phrase “dopamine” was entered in the Affymetrix NetAffx™ Analysis Center database (http://www.affymetrix.com/analysis/index.affx; accessed on 1 August 2021) to obtain probe names and identification numbers.

The expression profile of miRNAs in endometrial tissue samples was determined using GeneChip miRNA 2.0 microarrays (Affymetrix, Santa Clara, CA, USA), according to the manufacturer’s protocol. The GeneChip Scanner 3000 7G (Agilent Technologies, Santa Clara, CA, USA) was used to scan the microarrays. A TargetScan prediction tool (http://www.targetscan.org, accessed on 1 August 2021) was then used to determine which miRNAs differentiating endometrial cancer from the control could potentially affect the expression of dopamine-related mRNAs.

### 2.4. Real-Time Quantitative Reverse Transcription PCR

The expression profile of CXCL12, GNAL, OPRK1, DRD5, DRD3, DRD2, and COMT was determined by Real-Time Quantitative Reverse Transcription PCR (qRT-PCR) using SensiFast SYBR No-ROX One-Step Kit (Bioline, London, UK). β-actin (ACTB) was selected as endogenous control. 

The thermal profile included reverse transcription (45 °C, 10 min), polymerase activation (95 °C, 2 min), and 40 cycles involving denaturation (95 °C, 5 s), annealing (60 °C, 10 s), and elongation (72 °C, 5 s).

### 2.5. Enzyme-Linked Immunosorbent Assay (ELISA)

The protein level of CXCL12, OPRK1, DRD5, DRD2, DRD3, and COMT was assessed with following ELISA kits: Human CXCL12/SDF-1 alpha Kit (R&D Systems, Minneapolis, MN 55413, USA, Cat No. DSA00), Human Kappa Opioid Receptor Kit (MyBioSource, Inc., San Diego, CA, USA, Cat No. MBS3803118), Human Dopamine Receptor D5 Kit (MyBioSource, Inc., San Diego, CA, USA, Cat No. MBS724527), Human Dopamine Receptor D2 Kit (MyBiosource, Inc., San Diego, CA, USA, Cat No. MBS723432), Human Dopamine Receptor D3 Kit (MyBioSource, Inc., San Diego, CA, USA, Cat No. MBS722010), and Human Catechol-O-Methyltransferase (COMT) Kit (MyBioSource, Inc., San Diego, CA, USA, Cat No. MBS2019990).

### 2.6. Statistical Analysis

Transcriptome Analysis Console software (Thermo Fisher Scientific, Waltham, MA, USA) and Statistica 13.0 PL (Statsoft, Kraków, Poland) were used to perform statistical analysis. ANOVA and Tukey’s post hoc test were carried out (*p* < 0.05). Gene expression changes are presented as fold change (FC).

## 3. Results

### 3.1. Dopamine-Related Gene Expression Profile in Endometrial Tissues Determined by Microarrays and qRT-PCR

**Figure 1:**
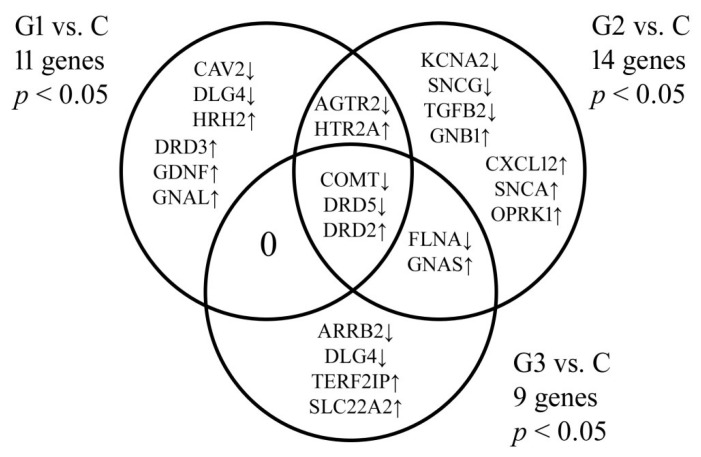
Venn diagram showing dopamine-related genes differentiating endometrial cancer from control. C, control, and G, endometrial cancer grade. *p* < 0.05 vs. C group.

A one-way ANOVA with Benjamini–Hochberg correction showed that among 175 dopamine-related mRNAs, the expression of 38 mRNAs representing 24 genes was significantly changed in endometrial cancer compared to control. Tukey’s post hoc test and a Venn diagram revealed genes characteristic of a given cancer grade or common to several groups (Figure 1).

Microarray analysis showed that DRD2 was overexpressed regardless of endometrial cancer grade, while COMT and DRD5 showed a significant decrease in expression. Changes in DRD3 levels along with GDNF, GNAL, HRH2, CAV2, and DLG4 were characteristic of G1 cancer. The results also revealed a decrease in KCNA2, SNCG, and TGFB2 levels with a simultaneous increase in GNB1, CXCL12, SNCA, and OPRK1 expression in G2 cancer compared to control. In the case of G3 cancer, significant reduction in ARRB2 and DLG4 levels and overexpression of TERF2IP and SLC22A2 were observed. AGTR2 and HTR2A were common to G1 and G2 cancer. In turn, FLNA and GNAS were common genes to G2 and G3 samples.

Then, in order to validate the microarray results, the expression profile of CXCL12, GNAL, OPRK1, DRD5, COMT, DRD2, and DRD3 was determined in endometrial tissues samples by qRT-PCR. Table 1 summarizes the results of both analyzes (*p* < 0.05).

The expression profile of the selected dopamine-related genes determined by the microarray technique was successfully validated by qRT-PCR. It has been observed that as endometrial cancer progresses, expression of CXCL12, GNAL, OPRK1, DRD2, and DRD3 increases while DRD5 and COMT levels are gradually reduced.

### 3.2. Dopamine-Related Proteins in the Serum of Patients Determined by ELISA

Expression of CXCL12, OPRK1, DRD5, COMT, DRD2, and DRD3 proteins was assessed in the serum of endometrial cancer patients and control group using ELISA (Table 2).

The analysis showed that as the grade of endometrial cancer increased, DRD5 and COMT levels decreased significantly. In turn, expression of CXCL12, OPRK1, DRD2, and DRD3 proteins increased with disease progression. The determined protein expression profile is consistent with the changes observed at the gene level.

### 3.3. Prediction of Dopamine-Related Gene Expression Regulation by miRNAs

Analysis with miRNA microarrays revealed miRNAs whose levels significantly changed in endometrial tissue samples compared to the control. Then, the use of TargetScan prediction tool allowed to match miRNAs to dopamine-associated mRNAs, expression of which was significantly altered in mRNA microarray experiment and confirmed by qRT-PCR and ELISA (Table 3).

It has been observed that miRNAs differentiating endometrial cancer from control are probably not involved in the reduction in COMT expression. In the case of DRD5, its low expression in endometrial cancer may be related to miR-15a-5p activity. High DRD2 levels may be due to silencing of miR-141-3p expression. In contrast, miR-4640-5p and miR-221-5p may participate in the regulation of DRD3 activity. In addition, overexpression of miR-135b-5p can be associated with a CXCL12 level increase.

## 4. Discussion

Cardiovascular disease and cancer are the leading causes of death worldwide. However, it is estimated that over the course of this century, cancer may become the leading cause of premature death in most countries [20]. For this reason, new diagnostic methods are being sought, including molecular markers enabling early cancer detection [21].

In this study, the expression profile of dopamine-related genes and proteins was determined. The dopaminergic system plays an important role in the survival of cancer cells, their invasion, and migration, which is possible due to the dopamine binding to its receptors [10]. Our results showed that changes in DRD1 and DRD4 levels were statistically insignificant. In contrast, DRD5 expression was decreased, while DRD2 and DRD3 levels were significantly increased in endometrial cancer. Signal pathways triggered by dopamine binding to D5 receptors cause activation of adenylyl cyclase, resulting in higher intracellular levels of cyclic adenosine monophosphate (cAMP). Leng et al. observed that activation of DRD5 in colon cancer, gastric cancer, and glioblastomas induced autophagic cell death [22]. Interestingly, DRD5 overexpression was observed in hepatocellular carcinoma [23]. In the case of endometrial cancer, Zhang et al. noticed a reduction in DRD2 and DRD5 expression in serous endometrial cancer after use of ONC206, which led to an inhibition of proliferation [24]. Reported in this study, reduction in DRD5 expression in endometrioid endometrial cancer samples could be the result of miR-15a-5p activity. Its level was elevated in the initial stage of the disease, and then it decreased significantly in G2 and G3 cancer. Kong et al. noticed that high expression of miR-15a-5p led to the promotion of cell proliferation and invasion in glioblastoma [25], which was also confirmed in ovarian cancer [26]. Interestingly, the studies conducted so far indicate that the level of miR-15a-5p was reduced both in the serum of patients [27] and in endometrial cancer cells and tissues [28]. Our results, therefore, may suggest that the interaction of this miRNA with DRD5 may play a role in the progression of endometrial cancer by regulating its proliferation.

DRD2 is an agonist of DRD5, and its high expression has been reported in many cancers, including breast cancer [29], ovarian cancer [30], and lung cancer [31]. In the case of DRD3, little is known about its involvement in tumor biology. Williford et al. observed that the level of D3 receptor is increased in glioblastoma and therapy with its antagonists may be promising [32]. Overexpression of DRD2 and DRD3 observed in our work was recorded at the gene and protein levels. Moreover, increased DRD2 levels may be the result of decreased miR-141-3p expression. Elevated levels of this miRNA leads to inhibition of proliferation in osteosarcoma cells [33], as well as migration and invasion of colorectal cancer [34]. Yang et al. also noted that high levels of miR-141-3p may be a risk factor in endometrial cancer [35]. This may suggest that DRD2 overexpression is due to the lack of regulatory effects of miR-141-3p. In addition, high DRD3 levels in endometrial cancer are associated with increased activity of miR-4640-5p and miR-221-5p, which are involved in tumor progression by regulating proliferation and metastasis [36,37].

Our analysis also showed an increase in the expression of kappa opioid receptor 1 (OPRK1), catechol-O-methyltransferase (COMT), and a decrease in the level of chemokine (C-X-C motif) ligand 12 (CXCL12). OPRK1 has the ability to inhibit the activity of adenylyl cyclase as well as the release of neurotransmitters [38]. In the case of cancer, its overexpression has been reported in neuroendocrine tumors [39]. Interestingly, the low level of OPRK1 was associated with a poor prognosis in hepatocellular carcinoma, while in lung cancer and melanoma it led to the growth of cancer cells [40]. Our results indicate that OPRK1 is overexpressed in endometrial cancer, which may be due to decreased miR-124-3p.1 levels. Inhibiting the activity of this miRNA promotes proliferation in bladder cancer [41], while in gastric cancer it also increases migration and metastasis [42].

COMT is involved in the degradation of catecholamines, including dopamine, and participates in estrogen metabolism [43]. This is especially important in the case of endometrial cancer, as it is largely estrogen dependent [44]. Estrogen is oxidized to catechol estrogens, which can damage DNA and have cancer potential. COMT catalyzes their methylation, leading to the formation of 2-methoxyestradiol, which has antiproliferative, apoptotic and cytotoxic properties [45]. Salama et al. observed that COMT knockdown in immortalized human endometrial glandular cells led to increased proliferation as well as induction of neoplastic transformation [46]. Similarly, Salih et al. reported that high progesterone levels increased COMT levels and, consequently, 2-methoxyestradiol, which resulted in proliferation inhibition in Ishikawa cells [47]. In this study, COMT expression was significantly decreased in endometrial cancer, both at the gene and protein levels. In addition, the COMT gene differentiated endometrial cancer samples from the control regardless of its grade, which indicates its potential utility as a complementary marker in endometrial cancer diagnostics. The observed reduction in COMT levels was most likely not a result of miRNA regulation.

CXCL12 binds to the chemokine (C-X-C motif) receptor 4 (CXCR4) and is involved in immune reactions as well as CNS development and neurotransmission. In the context of dopaminergic system, CXCL12 regulates migration and orientation of A9–A10 neurons [48]. In addition, CXCL12/CXCR4 signaling influences the orientation speed of dopaminergic neurons [49]. CXCL12 also plays an important role in cancer progression and metastasis, influencing overall survival in breast, lung, pancreatic, and esophagogastric cancer [50]. CXCL12-activated signaling cascades promote proliferation, survival, modulate cell adhesion, and migration, and regulate metastasis through the epithelial–mesenchymal transition. The pathway effectors include mitogen-activated protein kinases (MAPKs), phosphatidylinositol 3-kinase (PI3K), protein kinase B (Akt), and nuclear factor-κB (NF-κB) [51]. Gelmini et al. reported that the CXCL12/CXCR4 axis in HEC1A endometrial cancer line may promote its progression [52]. Similar conclusions were drawn by Liu et al. who observed continued secretion of CXCL12 in Ishikawa cells [53]. Cancer-associated fibroblasts derived from endometrial cancer also secrete CXCL12 and its high levels were associated with poor prognosis [54]. Interestingly, CXCL12 expression in estrogen receptor-negative endometrial cancer was associated with longer overall survival and recurrence-free survival, in contrast to estrogen receptor-positive cancer [55]. Overexpression of CXCL12 in endometrial cancer compared to the control observed in our study is consistent with previous reports. Moreover, levels of this chemokine increased with cancer grade and may be associated with high miR-135b-5β activity. The overexpression of miR-135b-5p promoted tumor cell survival and metastasis in gastric cancer [56], pancreatic cancer [57], and non-small-cell lung cancer [58], while in breast cancer the effect was the opposite [59]. High levels of miR-135b-5p have also been reported in endometrial cancer [60,61], however there has been no information about its association with CXCL12 so far.

The present study revealed the expression profile of dopamine-related genes and proteins in endometrial cancer, showing significant changes that may be associated with its progression. It is worth mentioning, however, that the limitation of this study was a small group of patients. Moreover, we focused on endometrioid endometrial cancer, which affects the vast majority of patients. Future studies on a larger group of patients and including other types of EC may be promising, as they may further expand the understanding of this cancer.

## 5. Conclusions

The overexpression of D2 and D3 receptors, with a simultaneous reduction in D5 receptor and COMT expression, indicates a disturbance in the functioning of the dopaminergic system. Moreover, it may be the result of miR-15a-5p, miR-141-3p, miR-4640-5p, and miR-221-5p activity, regulating proliferation or metastasis. In addition, decreased levels of miR-124-3p.1 may result in increased OPRK1 expression, contributing to endometrial cancer progression. Our results also confirmed the overexpression of CXCL12 observed in previous studies. Interestingly, it may be associated with high activity of miR-135b-5p, which promotes tumor cell survival and metastasis.

## Figures and Tables

**Table 1:** Dopamine-related gene expression profile in endometrial cancer determined by microarrays and qRT-PCR (*p* < 0.05).

ID	Gene	mRNA Microarrays			qRT-PCR		
		G1 vs. C	G2 vs. C	G3 vs. C	G1 vs. C	G2 vs. C	G3 vs. C
203666_at	CXCL12	8.59 *	10.02 *	11.45 *	9.54 *	10.36 *	12.54 *
206355_at	GNAL	5.08 *	7.77 *	8.98 *	5.14 *	6.98 *	9.14 *
206356_s_at	GNAL	4.98 *	7.54 *	9.01 *			
207553_at	OPRK1	2.14 *	9.52 *	15.08 *	1.52 *	8.41 *	16.36 *
208486_at	DRD5	−4.25 *	−3.69 *	−4.87 *	−4.65 *	−4.01 *	−4.30 *
208817_at	COMT	−8.54 *	−9.11 *	−9.66 *	−8.54 *	−9.74 *	−10.25 *
208818_s_at	COMT	−8.41 *	−9.36 *	−9.95 *			
211624_s_at	DRD2	8.41 *	12.36 *	14.99 *	8.41 *	12.65 *	15.47 *
216924_s_at	DRD2	8.47 *	12.25 *	15.03 *			
216938_x_at	DRD2	8.42 *	12.54 *	14.74 *			
211625_s_at	DRD3	10.25 *	11.98 *	19.58 *	10.66 *	12.54 *	21.99 *
214559_at	DRD3	10.33 *	12.06 *	21.01 *			

ID, number of the probe; C, control, and G, endometrial cancer grade. * *p* < 0.05 vs. C group.

**Table 2:** Serum dopamine-related protein expression profile in the study and control groups (*p* < 0.05).

Proteins (pg/mL)	Group			
	C	G1	G2	G3
CXCL12	654.21 ± 2.36	1452.36 ± 3.65 *	2987.25 ± 1.69 *	4874.6 ± 2.65 *
OPRK1	3.68 ± 0.65	4.96 ± 0.98 *	5.77 ± 1.14 *	8.01 ± 0.54 *
DRD5	4.58 ± 0.85	2.01 ± 0.74 *	0.89 ± 0.11 *	0.66 ± 0.36 *
COMT	3.11 ± 0.22	1.54 ± 021 *	0.88 ± 0.098 *	0.74 ± 0.14 *
DRD2	4.54 ± 0.55	8.14 ± 0.74 *	12.55 ± 1.33 *	14.09 ± 1.69 *
DRD3	4.66 ± 0.47	7.96 ± 0.98 *	11.54 ± 1.06 *	13.96 ± 1.11 *

C, control, and G, endometrial cancer grade. * *p* < 0.05 vs. C group.

**Table 3:** List of dopamine-related genes, whole activity may be regulated by miRNAs in endometrial cancer, determined with microarrays and TargetScan (*p* < 0.05).

mRNA	miRNA	miRNA Microarrays		
		G1 vs. C	G2 vs. C	G3 vs. C
*CXCL12*	hsa-miR−135b-5p	1.77 *	1.89 *	1.55 *
*OPRK1*	hsa-miR-124-3p.1	−2.01 *	−2.14 *	1.66 *
*DRD5*	hsa-miR-15a-5p	−1.01 *	4.74 *	2.01 *
*DRD2*	hsa-miR-141-3p	−2.69 *	−2.54 *	−3.01 *
*DRD3*	hsa-miR-4640-5p hsa-miR-221-5p	−3.01 * 2.51 *	1.02 * 2.77 *	1.44 * 1.25 *
*COMT*	-	-	-	-

C, control, and G, endometrial cancer grade. * *p* < 0.05 vs. C group.

## Data Availability

The data used to support the findings of this study are included in the article. The data will not be shared due to third-party rights and commercial confidentiality.
